# Lipid-Induced Adaptations of the Pancreatic Beta-Cell to Glucotoxic Conditions Sustain Insulin Secretion

**DOI:** 10.3390/ijms23010324

**Published:** 2021-12-28

**Authors:** Lucie Oberhauser, Pierre Maechler

**Affiliations:** Department of Cell Physiology and Metabolism, Faculty Diabetes Center, University of Geneva Medical Center, 1206 Geneva, Switzerland; Lucie.Oberhauser@unige.ch

**Keywords:** pancreatic islets, beta-cell, insulin, glucotoxicity, fatty acids

## Abstract

Over the last decades, lipotoxicity and glucotoxicity emerged as established mechanisms participating in the pathophysiology of obesity-related type 2 diabetes in general, and in the loss of β-cell function in particular. However, these terms hold various potential biological processes, and it is not clear what precisely they refer to and to what extent they might be clinically relevant. In this review, we discuss the basis and the last advances of research regarding the role of free fatty acids, their metabolic intracellular pathways, and receptor-mediated signaling related to glucose-stimulated insulin secretion, as well as lipid-induced β-cell dysfunction. We also describe the role of chronically elevated glucose, namely, glucotoxicity, which promotes failure and dedifferentiation of the β cell. Glucolipotoxicity combines deleterious effects of exposures to both high glucose and free fatty acids, supposedly provoking synergistic defects on the β cell. Nevertheless, recent studies have highlighted the glycerolipid/free fatty acid cycle as a protective pathway mediating active storage and recruitment of lipids. Finally, we discuss the putative correspondence of the loss of functional β cells in type 2 diabetes with a natural, although accelerated, aging process.

## 1. Introduction

### 1.1. Glucose-Stimulated Insulin Secretion in a Physiological Context

Insulin is the main anabolic hormone produced by a small subset of pancreatic cells, namely, the β-cells. Together with the glucagon-secreting α-cells and three other less abundant endocrine cell types (δ: somatostatin, γ: pancreatic polypeptide, and ε: ghrelin), β-cells cluster to form the islets of Langerhans. These islets, considered as mini-organs due to their abundant vascularization and innervation, are mainly nested in the tail of the pancreas and represent its endocrine part comprising less than 2% of this multifunctional organ. Islets of Langerhans control blood glucose homeostasis by finely regulating the secretion of both the hypoglycemic hormone insulin and the glucose-recruiting hormone glucagon. After a meal, the rise in blood glucose triggers insulin secretion from the β-cells, thereby promoting glucose uptake and glycogen stores in skeletal muscles and the liver, as well as fat storage in the adipose tissue. Concomitantly, insulin inhibits lipolysis in adipocytes and glucose production in the liver [1]. Altogether, these actions hold glycemia in the normal range (i.e., 4.5–6.9 mM). Conversely, under energy-demanding conditions, glucagon is secreted from the α-cells and promotes hepatic glucose production through glycogenolysis and eventually gluconeogenesis in order to maintain euglycemia [2].

**Figure 1:**
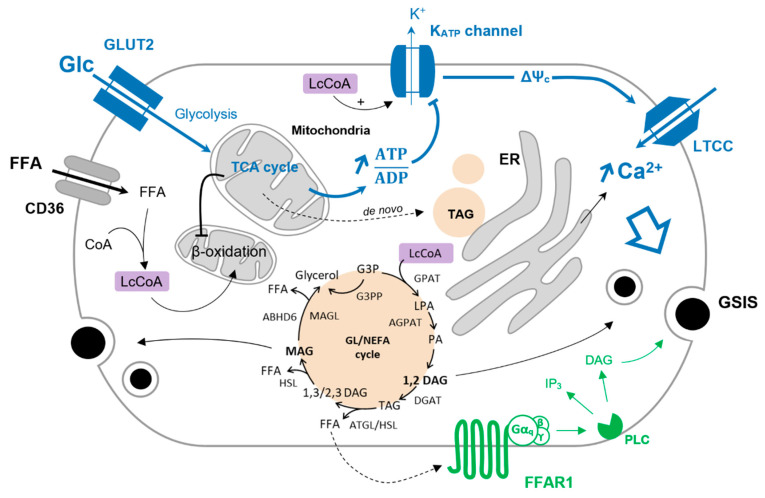
Glucose-induced FFA partitioning in the β-cell. Inhibition of β-oxidation by glucose (Glc) redirect Lc-CoA to the GL/NEFA cycle. Condensation of glycerol-trisphosphate and Lc-CoA is mediated by the glycerol-3-phosphate O-acyltransferase (GPAT) to form lysophosphatidic acid (LPA). LPA is further acetylated into phosphatidic by the 1-acyl-sn-glycerol-3-phosphate acyltransferase (AGPAT). The phosphohydrolase activity of lipins on PA produces 1,2-DAG that is then converted to TAG by the diacylglycerol O-acyltransferase (DGAT). TAG is sequentially hydrolyzed to 1/2,3-DAG, MAG, and finally glycerol by the adipose triglyceride lipase (ATGL), the hormone-sensitive lipase (HSL), and the MAG lipase (MAGL). MAG can also be hydrolyzed to glycerol by the α/β -hydrolase domain-containing 6 lipase (ABHD6—plasma membrane-bound enzyme). G3P can be directly converted to glycerol via the glycerol-3-phosphate phosphatase (G3PP) as a mechanism of gluco-detoxification. The triggering pathway of insulin secretion is shown in blue. Activation of FFAR1 further enhances insulin secretion, as shown in green.

Glucose-stimulated insulin secretion (GSIS) is a biphasic process [3]. The first phase is a direct consequence of the rise of glucose-induced intracellular calcium (i.e., the triggering pathway) and involves a readily releasable pool of insulin granules, which are already docked at the plasma membrane, allowing immediate insulin exocytosis [4]. Glucose is the chief nutrient triggering insulin secretion, and its sensing by the β-cells is mediated by the glucose transporter GLUT2 and the first rate-limiting enzyme of glycolysis, namely, glucokinase. As opposed to the ubiquitous hexokinase, glucokinase has a low affinity, yet high specificity, for glucose (K_m_ > 5 mM), conferring glucose sensor properties to the β-cells in the range of varying glycemia, thereby enabling insulin secretion only in response to elevated blood glucose concentrations [5,6]. Next, glycolysis generates pyruvate that is preferentially processed into the mitochondria, thanks to the minute amount of lactate being produced. The ATP generated by mitochondrial oxidative phosphorylation raises the cytosolic ATP to ADP ratio, promoting the closure of the ATP-sensitive potassium (K_ATP_) channels. The subsequent cell membrane depolarization enables calcium entry inside the cell via the L-type calcium channels (LTCC). The subsequent increase in cytosolic calcium triggers insulin granules exocytosis [7] (see Figure 1).

The second phase of GSIS develops gradually and refers to the amplifying pathway requiring additional coupling factors on top of permissive cytosolic calcium concentrations [8]. These factors do not initiate insulin secretion by themselves but potentiate GSIS via K_ATP_-independent mechanisms. Metabolism-derived factors of the amplifying pathway include nucleotides, such as NADPH and ATP, and metabolites—in particular, glutamate, long chain acyl-CoA (Lc-CoA), and other lipid metabolism derivatives that have been described [9,10]. Interestingly, some of these factors may also exert autocrine or paracrine signaling. For instance, ATP release may activate P2Y purinergic receptors [11], and the export of free fatty acids (FFA) can result in the activation of their respective G-protein-coupled receptors [12], enhancing the secretory response. Conversely, glutamate secreted by the islets exert negative feedback on insulin secretion by targeting N-methyl-D-aspartate (NMDA) receptors on the β-cell, thereby promoting K_ATP_ channel re-opening and a decrease in calcium influx lowering insulin secretion [13]. Among other extracellular signaling molecules, incretins (GIP, GLP-1) secreted by the enteroendocrine cells potentiate GSIS [14,15].

### 1.2. Role of Fatty Acids in the Potentiation of GSIS

Food is the major source of lipids and more specifically of poly-unsaturated fatty acids (PUFA) as they cannot be synthetized by humans who lack specific desaturases. Triglycerides are converted to long-chain fatty acids (Lc-FA, C > 12) by lipases of the digestive juices releasing, among others, palmitate (C16:0, saturated), oleate (C18:1, mono-unsaturated), and linoleate (C18:2, poly-unsaturated). Short-chain fatty acids (Sc-FA, C < 6) are mainly produced in the colon by the gut microbiota as the result of fermentation of the undigested carbohydrates [16]. Sc-FA are essentially signaling molecules targeting the FFA receptors FFAR2 and FFAR3 (formerly referred to as GPR43 and GPR41, respectively). They recently triggered attention for their potential role in the regulation of the gut–brain axis, in glucose and lipid homeostasis, as well as inflammation and the immune response [17]. Lc-FA fulfill a wider variety of biological functions [18]. They constitute the major membrane component of cells and organelles under the form of phospholipids. After activation by coenzyme-A (CoA), they also serve as energy providers through mitochondrial β-oxidation. Finally, they act as signaling molecules both indirectly through metabolic derivatives, such as ceramides, and directly through the activation of membrane receptors FFAR1 and FFAR4 (or GPR40 and GPR120, respectively) [19].

The β-cells express both Sc-FA and Lc-FA receptors. The role of these receptors in β-cell function started to be investigated about 20 years ago after the FFAR1 was deorphanized by Briscoe et al. in 2003 [20]. They showed that medium to Lc-FA target FFAR1, which involves Gα_q_ signaling and is mainly expressed in β-cells and human brain. In the same year, Itoh et al. uncovered the role of FFAR1 in the acute potentiation of GSIS through the elevation of intracellular calcium [21]. Soon, FFAR1 appeared as a potential therapeutic target for the treatment of type-2 diabetes, resulting in intensive, although somehow disappointing, research for the development of corresponding agonists.

While Sc-FA receptors were deorphanized at the same period as their Lc-FA counterparts [22], the study of their functions was a little left behind until the recent awareness of the importance of the microbiota in metabolic diseases. To date, the presence of FFAR2 and FFAR3 in β-cells is well documented but their role in the regulation of insulin secretion remains controversial. The natural ligands acetate or propionate have marginal effects on GSIS [23,24,25], while FFAR2 mediates the potentiating effect of these Sc-FA on the glucose response of isolated islets [25,26]. Regarding synthetic agonists of FFAR2, phenylacetamide (PA, compound 58) enhances GSIS in human islets [24], whereas 4-CMTB inhibits GSIS in human pseudo-islets [23]. In mouse models, upregulation of FFAR3 impairs GSIS and glucose tolerance, while its global knockout improves both parameters [27]. The study of islets isolated from the global FFAR3 knockout mice pointed to an inhibitory effect of FFAR3 signaling upon GSIS, an effect mediated by Gα_i_ activation and subsequent lowering of cAMP production [28]. Regarding FFAR2, its global knockout does not affect glucose homeostasis of the mice, although GSIS is impaired in vitro [25]. On a high-fat diet, mice lacking FFAR2 exhibit impaired glucose tolerance along with reduced GSIS [24]. In another study, researchers deleted both FFAR2 and FFAR3 (global and β-cell specific knockouts, respectively) resulting in an increase of insulin secretion and the improvement of glucose tolerance in mice fed a high-fat diet [29]. Finally, it has been reported that in vitro activation of FFAR2 protects human islets from apoptosis induced by cytokines [26]. Given the recent interest for the Sc-FA receptors in the β-cell function, more studies are needed to determine their exact role in insulin secretion.

### 1.3. FFAR1 Signaling in GSIS

Among the four members of the FFAR family, FFAR1 has been the most extensively studied in the β-cell. The mechanism by which FFA acutely potentiate GSIS through FFAR1 involves Gα_q_ signaling that activates phospholipase C (PLC) that in turn hydrolyzes phosphatidylinositol-4,5-bisphosphate (PIP_2_) into inositol trisphosphate (IP_3_) and diacylglycerol (DAG). IP_3_ targets its receptors at the endoplasmic reticulum (ER) membrane, promoting calcium release from ER stores and resulting in the further elevation of cytosolic calcium primarily initiated by the glucose stimulation [30] (see Figure 1). DAG derived from PIP_2_ hydrolysis may also directly participate in the potentiation of GSIS by targeting PKC and modulating insulin granule exocytosis [31,32].

Natural ligands of FFAR1 comprise medium and Lc-FA. Since its identification, more than 20 synthetic FFAR1 ligands were discovered by academic groups and pharmaceutical companies, none of them ultimately ending up in therapeutic use [33]. The analysis of the crystal structure of FFAR1 bound to the agonist TAK875, as well as functional assays, allowed for the unravelling of new binding pockets and multiple allosteric sites for FFAR1 ligands [34,35]. Furthermore, although natural ligands signal through Gα_q_, some synthetic agonists are able to recruit Gα_s_, thereby activating adenylate cyclase and increasing cAMP production [36]. In parallel, the concept of biased agonism elicited by FFAR1 ligands was raised on the basis of the observation of β-arrestin and Gα_q_ signaling being differentially activated by FFAR1 ligands, while being both involved in the insulinotropic activity [37]. These conclusions highlight the complexity of FFAR1 signaling in particular and of GPCR signaling in general, opening encouraging perspectives for the development of novel selective anti-diabetic drugs.

## 2. Glucose-Stimulated Insulin Secretion in a Pathophysiological Context

### 2.1. Pathophysiology of the β-Cell

Failure in maintaining a critical and functional β-cell mass leads to the development of diabetes. Destruction of the β-cells with detection of auto-antibodies is a hallmark of type 1 diabetes characterized by early onset of the disease and immediate insulin dependency, while late onset type 2 diabetes is primarily associated with insulin resistance and a progressive loss of β-cell function [38]. Recently, the classification criteria of the disease have been revised according to the diversity of diabetic subgroups [39]. Whether caused by genetic or environmental factors, or both, diabetic patients all share a common feature that is hyperglycemia, i.e., fasting blood glucose above 7 mM [38]. According to the World Health Organization, 422 million adults suffer from diabetes, which represents 1 person out of 11, twice as much as in 1980. This progression correlates with an increase in the incidence of overweight (BMI 25–30 kg/m^2^) and obesity (BMI > 30 kg/m^2^) and, although not all obese individuals develop diabetes, it represents the main risk factor for type-2 diabetes [40]. A feature frequently observed in obese subjects is a feeding behavior with frequent meals of energy-rich processed food and sweet beverages. This favors chronic elevated blood nutrients levels, mainly glucose and lipids, promoting insulin resistance and potentially impairing β-cell function.

The human body copes with excess feeding by increasing insulin secretion to promote global nutrient storage, in particular fat depots. Obese individuals may develop insulin resistance and/or glucose intolerance without ever becoming diabetic as long as they compensate by secreting more insulin and expand a non-deleterious adipose tissue [41]. However, the system may saturate, leading to adipose tissue dysfunction and lipid spillover [42]. This results in the release of non-esterified fatty acids (NEFA) and the secretion of pro-inflammatory adipokines in the bloodstream, eventually leading to ectopic fat deposit, notably in the liver [43,44,45,46,47]. Accumulation of hepatic lipids promotes liver dysfunction and inflammation, a condition known as non-alcoholic fatty liver disease (NAFLD), which can further evolve toward steatosis, cirrhosis, and ultimately liver cancer [48,49]. Inflammation of metabolic organs (liver, adipose tissue, muscles) promotes their resistance to insulin, thereby contributing to increased insulin secretion by the β-cell as a compensatory mechanism. In the meantime, the β-cell is overwhelmed with circulating NEFA released by the adipocytes, including from neighboring fat cells [50], as well as with glucose produced by the liver as a consequence of impaired insulin signaling. Altogether, these factors promote glucose intolerance and hyperglycemia. The effects of chronic elevated glucose and fatty acids on β-cell function have been greatly investigated over the past decades, giving rise sequentially to the concepts of glucotoxicity, lipotoxicity [51], and finally glucolipotoxicity [52].

### 2.2. Glucotoxicity

The maintenance, by diabetic patients, of glycaemia within a 4–10 mM range is critical and challenging. This may lead to alternation of hypoglycemic and hyperglycemic phases with dramatic complications. Hyperglycemia has long-term deleterious consequences, notably affecting microvessels and nerves, leading to nephropathies, neuropathies, and retinopathies, as well as higher cardiovascular risk [53,54,55]. Because hypoglycemia has severe immediate effects, such as attention deficit, convulsion or even coma, the glucose-lowering strategies of diabetic patients are often suboptimal, allowing slightly elevated glycaemia. In this context, understanding the effects of chronic elevated blood glucose on β-cell identity and function is of major importance.

A third major process that is thought to be the main contributor to the loss of functional β-cell mass induced by recurrent hyperglycemia is β-cell dedifferentiation. Several in vitro and in vivo studies showed that chronic elevated glucose levels reduce expression of β-cell-specific transcription factors such as *Pdx1/IPF1*, *MafA*, or *Nkx-6.1*, resulting in β-cell reversion to progenitor-like cells or transdifferentiation to α-cells [56,57,58,59,60]. Beyond transcription factors, glucotoxicity affects the expression of several genes involved in mitochondrial function [61], energy sensing [62], glucose and lipid metabolism, and exocytosis, collectively converging towards β-cell dysfunction and/or death [63,64]. Of note, upregulation of UCP2 as a stress response confers protective effects against glucotoxic conditions and oxidative stress [65]. The function of UCP2 in normal β-cells remains unclear and somehow controversial as its knockout in mice results in improved or impaired GSIS when animals are on a mixed versus congenic background, respectively [66,67]. Glucotoxicity also promotes inflammation, notably by increasing the production of IL-1β that activates NF-κB altering β-cell function [68]. Moreover, elevated pro-inflammatory cytokine levels contribute to β-cell dedifferentiation through *Foxo1* downregulation [69].

### 2.3. Lipotoxicity

Lipotoxicity is considered as a major triggering factor in the onset of type-2 diabetes by promoting tissue inflammation and insulin resistance of peripheral tissues [47,70]. In addition, it was proposed to further contribute to the development of the disease by directly altering β-cell function and integrity [51], although this aspect has been challenged recently [71].

Studies on lipotoxicity mainly focus on the effects of palmitate and oleate on β-cell viability. In this regard, the saturated fatty acid palmitate promotes β-cell death via two main mechanisms, i.e., ER stress and ceramide formation. The former has been largely documented by M. Cnop and colleagues. According to their studies, palmitate would induce apoptosis by promoting the depletion of ER calcium stores, leading to the activation of the IRE1, PERK, and ATF6 proteins of the UPR pathway [72,73]. The unsaturated fatty acid oleate would also activate this pathway, although to a lesser extent, suggesting a NEFA-specific degree of toxicity. This observation is in contradiction with results also obtained in rat insulinoma cells by Karaskov et al. who reported that, as opposed to palmitate, oleate-induced apoptosis is independent of the UPR pathway [74]. Adding complexity to these lipotoxic mechanisms, Marmugi et al. showed that sorcin, a calcium-binding protein that mediates termination of calcium-induced calcium release, maintains ER calcium stores and preserves GSIS [75]. As shown both in vitro and in vivo, sorcin expression is decreased in β-cells and human islets chronically exposed to palmitate as well as in islets from mice fed a high-fat diet composed mainly of saturated fat, stressing the role of palmitate triggering ER stress and potentially apoptosis [75]. An important player in ER calcium homeostasis is the pump SERCA (sarcoendoplasmic reticulum pump Ca^2+^ ATPase). Although cytokines have been shown to downregulate SERCA2b in β-cells, thereby promoting ER stress and apoptosis [76], such an effect has not been ascribed to lipotoxicity. Several other pathways associated with palmitate-induced ER stress were investigated, notably the aberrant palmitoylation of some proteins [77] or the increase in ROS production following fatty acids exposure [78]. Regarding the latter, mitochondria appear to be a primary source of ROS upon fatty acid exposure and, at the same time, targets for ROS [79]. In human islets, this is associated with changes of some key components of the mitochondrial machinery [63].

Palmitate is a precursor for ceramide biosynthesis. Chronic palmitate treatment substantially increases cellular ceramide levels that have been linked to β-cell dysfunction and apoptosis [80,81]. More precisely, Manukyan et al. showed that ceramide levels are doubled in MIN6 cells and human islets following palmitate exposure due to increased expression of palmitoyl transferase and ceramide synthases [81]. In their study, although palmitate induced the synthesis of ceramides through both the de novo and the salvage pathways, only the latter contributed to reduced β-cell function and viability. The mechanisms by which ceramides induce apoptosis are not well understood, with some studies pointing to a direct link with ER stress and mitochondria permeabilization [80,82]. The changes in intracellular lipid composition following chronic exposure to NEFA may also impact on membrane and organelle structures. Indeed, INS-1E β-cells cultured with palmitate or oleate exhibit lower membrane tension, revealing altered physicochemical properties of the cells [83].

Upon lipotoxic conditions, mono- and poly-unsaturated fatty acids in general, and oleate in particular, have been assigned some protective properties on β-cells by preventing deleterious effects of saturated fatty acids, mainly palmitate [81,84,85]. In contrast to high glucose, chronic exposure to fatty acids does not induce major changes in gene expression or dedifferentiation in INS-1E β-cells as well as in human islets [86]. However, in rat islets, it was observed that chronic palmitate decreases *MafA* expression and alters PDX-1 nuclear localization [87].

Lipotoxicity contributed by chronic fatty acids could also be induced through signaling upon binding to their receptors. The role of FFAR1 (or GPR40) in the acute potentiation of GSIS by exogenous NEFA has been well described [21,88,89]. However, its putative role in lipotoxicity gave rise to some controversies. Steneberg et al. presented seminal evidence for a role of FFAR1 in the deleterious effects of chronic fatty acids on β-cells [90]. They showed that *Ffar1* knockout mice are notably protected against obesity-induced hyperinsulinemia and glucose intolerance. Conversely, β-cell-specific overexpression of *Ffar1* impairs β-cell function. Several other in vivo and in vitro studies reported similar effects, although inconsistencies emerged regarding specific aspects of *Ffar1* knockout mice [91,92,93,94]. For instance, Latour et al. observed no difference in glucose homeostasis between *Ffar1* knockout and wild type mice, and their isolated islets were similarly sensitive to fatty acid inhibition of GSIS following 3 days of exposure to palmitate or oleate [95]. Similarly, Lan et al. reported no difference between control and *Ffar1* null mice fed either a standard chow diet or a high-fat diet [96], and the double knockout of *Ffar1* and *Ffar4* does not change the glucose tolerance in mice fed a high-fat diet, as recently shown by Croze et al. [97]. Overexpression of the human *FFAR1* in β-cells enhances insulin secretion and improves glucose tolerance both in mice fed a high-fat diet and in diabetic KK mice [98]. Regarding β-cell loss upon fatty acid exposure, a couple of studies reported that FFAR1 signaling protects against palmitate-induced β-cell death [99,100]. Collectively, these results emphasize the difficulty to delineate the exact role of FFAR1 in the response of β-cells to lipotoxicity. In the absence of reliable antibodies, FFAR1 studies suffer from poor *Ffar1* characterization at the protein level preventing the assessment of proper controls [101].

Besides their bona fide FFAR receptors, NEFA may trigger an inflammatory response through their binding to toll-like receptors of the innate immune system present on β-cells and on neighboring macrophages. This induces activation of NF-kB and the production of proinflammatory cytokines, such as IL-1β [102]. Alike the FFAR1 response, acute versus chronic activation of this immune pathway may result in the potentiation or the alteration of GSIS, respectively [103].

### 2.4. Glucolipotoxicity

Phenotypically, lipotoxicity and glucotoxicity are closely interconnected. In a context of obesity-induced type-2 diabetes, high glucose promotes fat deposit and elevated circulating NEFA levels alter glucose clearance. The paradigm of glucolipotoxicity states that the effects of glucotoxicity and lipotoxicity are not simply additive but rather impair organ function in a synergistic way. It was also suggested that some lipotoxic effects are induced only in the presence of elevated glucose concentrations because glucose determines intracellular fatty acid partitioning [52,104,105]. These observations gave rise to the concept of glucolipotoxicity, although its relevance at the clinical level is not firmly established [71].

In the mid-nineties, M. Prentki and B. Corkey introduced the concept of “glucolipoxia” postulating that in obesity-related type-2 diabetic patients the different tissue-specific defects share the same metabolic traits, i.e., elevated malonyl-CoA and Lc-CoA [52,106]. In this view, high glucose metabolism increases intracellular malonyl-CoA levels inhibiting fatty acid β-oxidation, thereby favoring accumulation of Lc-CoA in the cytosol [105]. This model was supported by the inhibition of β-oxidation in INS 832/13 cells promoting palmitate-induced β-cell apoptosis, whereas AICAR lowered glucolipotoxicity by redirecting Lc-CoA toward β-oxidation. The same study highlighted a fatty acid specific signature regarding the synergism with high glucose on β-cell death. Indeed, the association of chronic elevated glucose with palmitate and stearate induced a marked synergistic effect on apoptosis, while the association with linoleate and oleate had milder and null synergistic effects, respectively. These results revealed that, in the β-cell, lipotoxicity depends on glucotoxicity and that the cooperative effects of both, namely, glucolipotoxicity, relies on the fatty acid nature.

Although the detailed mechanisms underlying glucolipotoxicity remain unclear, they appear to be mediated by several intracellular metabolites affecting different cellular pathways [107]. Among them, the previously described ER stress and the generation of ceramides contribute to the toxic effects of chronic elevated glucose combined with fatty acids on β-cells. For instance, INS-1 cells cultured with palmitate and high glucose exhibit ceramide trafficking between ER and Golgi, forcing ceramide accumulation in the ER and thus promoting ER stress [108]. Glucolipotoxicity also promotes a β-cell inflammatory response by further activating NF-κB through the upregulation of TNFRSF5 [109].

**Figure 2:**
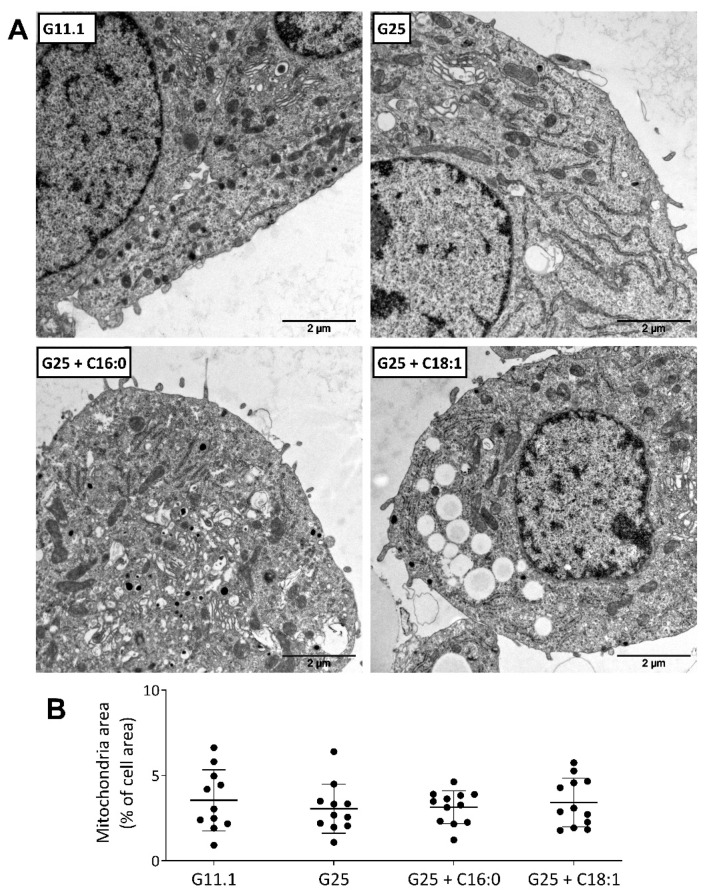
Ultrastructure of INS-1E β-cells following glucolipotoxic culture conditions. Cells were exposed to standard 11.1 mM (G11.1) and high 25 mM (G25) glucose concentrations without (BSA) or with 0.4 mM palmitate (C16:0) or oleate (C18:1) for 3 days. (**A**) Representative electron micrographs of cells cultured at G11.1 and G25 plus C16:0 and C18:1, scale bar 2 µm. (**B**) Quantitative analysis of intracellular area occupied by mitochondria. Results are presented as means ± SD, *n* = 11–12.

Regarding ultrastructural changes induced by glucolipotoxic conditions, mitochondria have been reported to be sensitive to high nutrient exposure. Different hyperglycemic animal models present mitochondrial alterations in the β-cells, reviewed in [67], and in type-2 diabetic patients, islet mitochondrial abnormalities have been observed [110]. In INS-1 cells, mitochondrial dynamics controlled by continuous fusion and fission events are balancing toward higher degree of fragmentation when exposed to high glucose and palmitate [111]. Using electron microscopy, we investigated the morphology and density of mitochondria in INS-1E cells following chronic exposure to high glucose and palmitate versus oleate. Three days of culture in glucolipotoxic conditions did not modify the overall cellular occupancy of mitochondria (Figure 2). Of note, the association of high glucose with oleate, much more than palmitate, promoted massive accumulation of lipid droplets in INS-1E β-cells [86].

## 3. The Glycerolipid/NEFA Cycle

### 3.1. Functioning of the Glycerolipid/NEFA Cycle

Glucose is the main coordinator of β-cell function, and its metabolism governs the metabolic fate of fatty acids. Upon glucose deprivation, cytosolic fatty acids activated in the form of Lc-CoA can be funneled into mitochondria toward β-oxidation to ensure sufficient energy supply supporting basal cell functions (Figure 1). A rise in blood glucose levels increases glucose-derived pyruvate and the activity of the TCA cycle independently of the energy requirement when it comes to the β-cell. This results in the production of citrate that escapes the mitochondria and activates the acetyl-CoA carboxylase (ACC) to generate cytosolic malonyl-CoA. In turn, malonyl-CoA inhibits the carnitine palmitoyltransferase I (CPT1), blocking the entry of Lc-CoA into mitochondria and disabling β-oxidation [112,113]. In this context, Lc-CoA are redirected towards other pathways, notably to the glycerolipid/NEFA cycle (Figure 1). Originally, this cycle was described in the adipocytes as a mechanism to promote lipid accumulation under the form of triacylglycerol (TAG or triglycerides) and was thought to be marginal in other cell types, in particular β-cells. However, with the deeper understanding of the mechanisms underlying GSIS and its amplifying pathway, the glycerolipid/NEFA (GL/NEFA) cycle appeared as a candidate for a source of metabolic intermediaries involved in the potentiation of insulin secretion.

### 3.2. The GL/NEFA Cycle in a Physiological Context

The GL/NEFA cycle is composed of lipogenic and lipolytic arms (Figure 1). Lipogenesis involves the consecutive formation of lysophosphatidic acid (LPA), phosphatidic acid (PA), DAG, and finally TAG from glycolysis-derived glycerol-3-phosphate (G3P) and Lc-CoA. The lipolysis arm of the cycle breaks down TAG to DAG and MAG, ultimately releasing glycerol [114]. As β-cells barely express glycerol kinase, glycerol leaves the cell through aquaglyceroporins [115,116]. Enzymes catalyzing lipogenesis and lipolysis reactions are not fully described but consist principally of redundant and compartmentalized acyltransferases (GPAT, AGPAT, DGAT) and lipases (ATGL, HSL, MAGL/ABHD6) [114,117]. As shown in INS-1E cells and human islets, glucotoxic conditions upregulate genes encoding enzymes of the GL/NEFA cycle [86], underlying the interplay between high glucose and intracellular lipid turnover.

Glucose stimulation induces lipogenesis by redirecting NEFA to esterification and by favoring de novo lipid synthesis [118]. In the β-cell, the latter pathway is marginal due to low expression of fatty acid synthase [106], and paradoxically, glucose promotes lipolysis [19,119,120,121]. This suggests that GL/NEFA cycling is implicated in β-cell activity. Mulder et al. first substantiated the role of the GL/NEFA cycle in β-cell function by showing that inhibition of lipolysis by the pan lipase inhibitor Orlistat impairs GSIS [122]. A few years later, Fex et al. showed that mice lacking HSL in the β-cells exhibit altered GSIS [123] and Attané et al. demonstrated that β-cell-specific ATGL knockout mice have lower insulinaemia and GSIS [124]. Collectively, these results underscore the role of the GL/NEFA cycle in general and its lipolytic arm in particular for proper β-cell function.

Intermediates of lipid metabolism, mainly from the GL/FFA cycle, have been put forward as coupling factors in GSIS. However, their exact nature and the mechanisms by which they modulate GSIS remain unclear [114]. Among those metabolites, DAG and MAG have attracted much of the attention. DAG is formed either during lipogenesis from PA or upon lipolysis from direct hydrolysis of TAG. According to the specificity of the biochemical reactions, different species of DAG may be produced. Lipin-mediated PA phosphohydrolase generates 1,2-DAG, while the hydrolysis of TAG by ATGL produces either 1,3-DAG or 2,3-DAG [125,126]. Among these DAG species, only 1,2-DAG qualifies as a signaling molecule by activating PKC, which further phosphorylates target proteins of the exocytotic machinery and induces actin remodeling [32]. Regarding MAG, it was shown to promote insulin granule exocytosis by binding to the exocytotic protein Munc13-1 [127,128]. The role of MAG in β-cells has been substantiated in ATGL knockout mice where exogenous MAG supply restores GSIS [124].

### 3.3. GL/NEFA Cycling in Type-2 Diabetes

Obesity may lead to β-cell dysfunction and potentially diabetes through the mechanisms of glucolipotoxicity described above. Potential β-cell failure is preceded by a compensatory phase during which β-cell mass and insulin secretory capacity are increased in order to compensate for obesity-induced insulin resistance [107,129]. Specifically, it was suggested that, in this context, GL/NEFA cycling is enhanced as an adaptive response [114]. When islets isolated from obese Zucker-Fatty rats are acutely exposed to stimulatory glucose and palmitate, GSIS is increased, along with NEFA esterification and lipolysis, while lipase inhibition reduces insulin secretion [130]. In the compensatory phase, enhanced GL/NEFA cycling would promote higher β-cell secretory capacity by increasing the production of coupling factors such as DAG and MAG. However, it has also been proposed by Prentki and colleagues that this cycle could serve as a path for excess fuel detoxification [114]. Indeed, converted to glycerol part of surplus glucose is no longer metabolized and leaves the β-cell through aquaglyceroporins, relieving the cell from some of the glucotoxic effects. The identification of a glycerol-3-phosphate phosphatase (G3PP) that converts G3P to glycerol without engaging the whole GL/NEFA cycle is supportive of this glycerol-mediated gluco-detoxification [131]. Similarly, esterification of NEFA from Lc-CoA and glycerol-3-phosphate into TAG would promote lipo-detoxification by preventing the formation of harmful lipid derived species.

The exact role of the GL/NEFA cycle in β-cell glucolipotoxicity remains unclear, and its contribution to the development of type-2 diabetes lacks in vivo evidence. In rodent models, it was observed that lipolysis and NEFA esterification were reduced in high-fat diet-induced obese mice exhibiting early and pre-diabetes, suggesting alteration of the GL/NEFA cycle already in the pre-diabetic stage [132]. Several other studies reported unbalanced GL/NEFA cycle in rodent models of type-2 diabetes, notably increased lipogenesis accompanied by reduced or unchanged lipolysis [114]. On the basis of studies in human islets and INS-1E cells, we recently reported that the GL/NEFA cycle plays a central role in the preservation of GSIS upon glucolipotoxic culture conditions [86]. Such a protective effect requires acute intracellular TAG mobilization, thanks to the adaptive response of GL/NEFA cycle gene expression [86].

## 4. Discussion

The concept of glucolipotoxicity emerged at the turn of the millennium and has given rise to numerous studies. After all these years, we still lack convincing explanation for the loss of functional β-cells in obese patients who develop type-2 diabetes. Most of the published investigations have been conducted thoroughly. So why such discrepancies? With its multifactorial components, the core of the disease itself could account for the various outcomes associated with specificities of study design.

Aging is associated with a progressive decline in insulin secretion, independently of the BMI and insulin sensitivity. This has been reported in human subjects, both in vivo on different populations and in vitro on islets isolated from donors [133]. The decline is typically observed in subjects aged >60 years. Interestingly, there is no correspondence in rodents with their lifespan of a couple of years [133], indicating that time per se is a component of the damaging mechanisms. In humans, a gradual loss of functional β-cells, at slow pace over a lifetime, appears as a natural process. In view of the various and complex etiologies proposed thus far to account for the development of type-2 diabetes, one can hypothesize that such a pathophysiological development might actually represent an accelerated aging process. Over the last decades, with the rapid rise in the incidence of type-2 diabetes affecting younger subjects, the classification of the disease evolved from adult-onset diabetes to noninsulin-dependent diabetes mellitus (NIDDM) and then type-2 diabetes (https://www.who.int/publications/i/item/classification-of-diabetes-mellitus) (accessed on 6 December 2021). This reflects the much earlier onset of the disease with an accelerated progression eventually leading to the requirement of exogenous insulin. As a result, type-2 diabetes is no longer primarily associated with elderly people. In other words, obesity could favor accelerated aging of the β cells by exposing this hardly renewable material to chronically elevated levels of trivial metabolic stresses such as glucose, fatty acids, cytokines, and reactive oxygen species. If correct, this concept could reconcile some discrepancies found among studies reporting various degrees of damaging effects of the stress candidates on β-cell function.

## 5. Conclusions

Over the last decades, the nomenclature around type-2 diabetes has been enriched with some appealing concepts translated into suitcase words such as diabesity, lipotoxicity, glucotoxicity, or glucolipotoxicity. Although these terms are catchy and can easily convey a straightforward message, the scientific evidence for their clinical relevance is weak. In particular, the suffix toxicity associated with a nutrient is misleading. If most of the studies agree on the deleterious consequences of chronic high glucose, rather than glucose per se, the supposedly toxic effects of NEFA are less convincing. The recruitment of stored lipids might be beneficial if used for pathways supporting cell function. Accordingly, the dynamic and funneling of NEFA, rather than their absolute levels, appear as central for our comprehension of the complex interactions of metabolites with β-cell function.

## 6. Materials and Methods

### 6.1. Reagents

D-glucose, fatty acids, culture media, and other basic reagents were obtained from Sigma-Aldrich (St.-Louis, MO, USA).

### 6.2. Cell Culture and Treatments

Rat INS-1E β-cells [134] were grown in RPMI-1640 medium at 11.1 mM glucose supplemented with 5% (*v*/*v*) heat-inactivated fetal calf serum (FCS). At 4 days after seeding, cells were further cultured for 3 days at either 11.1 mM glucose (G11.1, control) or exposed to high 25 mM (G25) glucose concentrations. INS-1E β-cells were also exposed to 0.4 mM palmitate or 0.4 mM oleate in the presence of 0.5% BSA, as detailed previously [56,83].

### 6.3. Electron Microscopy

INS-1E beta-cells treated for 3 days with different glucose concentrations and fatty acids were fixed with 2% glutaraldehyde in 0.1 M pH 7.4 phosphate buffer for 1 h at RT. Cells were scrapped, washed, and pelleted in phosphate buffer. Samples were further dehydrated, epon-embedded, and cut at the faculty electron microscopy core facility (PFMU, University of Geneva). Images were acquired on the Morgagni transmission electron microscope (FEI Company, Eindhoven, The Netherlands).

## Data Availability

Raw data generated for original results presented in this study are available from the corresponding author upon reasonable request.
